# Loggerhead Sea Turtles as Hosts of Diverse Bacterial and Fungal Communities

**DOI:** 10.1007/s00248-024-02388-x

**Published:** 2024-05-30

**Authors:** Klara Filek, Borna Branimir Vuković, Marta Žižek, Lucija Kanjer, Adriana Trotta, Antonio Di Bello, Marialaura Corrente, Sunčica Bosak

**Affiliations:** 1 Department of Biology, Faculty of Science, University of Zagreb, Horvatovac 102a, HR-10000 Zagreb, Croatia; 2 Ruđer Bošković Institute, Bijenička 54, HR-10000 Zagreb, Croatia; 3 Campus Universitario, University of Bari “Aldo Moro”, Via Orabona 4, 70125 Bari, BA Italy; 4 Department of Veterinary Medicine, University of Bari “Aldo Moro”, Str. Prov. Per Casamassima Km 3, 70010 Valenzano, BA Italy; 5 Diagnostic and Research Institute of Hygiene, Microbiology and Environmental Medicine, Medical University of Graz, Neue Stiftingtalstraße 6, 8010 Graz, Austria

**Keywords:** *Caretta caretta*, Wild microbiome, Reptile microbiota, Conservation efforts, Mycobiota

## Abstract

Research on microbial communities associated with wild animals provides a valuable reservoir of knowledge that could be used for enhancing their rehabilitation and conservation. The loggerhead sea turtle (*Caretta caretta*) is a globally distributed species with its Mediterranean population categorized as least concern according to the IUCN Red List of Threatened Species as a result of robust conservation efforts. In our study, we aimed to further understand their biology in relation to their associated microorganisms. We investigated epi- and endozoic bacterial and endozoic fungal communities of cloaca, oral mucosa, carapace biofilm. Samples obtained from 18 juvenile, subadult, and adult turtles as well as 8 respective enclosures, over a 3-year period, were analysed by amplicon sequencing of 16S rRNA gene and ITS2 region of nuclear ribosomal gene. Our results reveal a trend of decreasing diversity of distal gut bacterial communities with the age of turtles. Notably, *Tenacibaculum* species show higher relative abundance in juveniles than in adults. Differential abundances of taxa identified as *Tenacibaculum*, *Moraxellaceae*, *Cardiobacteriaceae*, and *Campylobacter* were observed in both cloacal and oral samples in addition to having distinct microbial compositions with *Halioglobus* taxa present only in oral samples. Fungal communities in loggerheads’ cloaca were diverse and varied significantly among individuals, differing from those of tank water. Our findings expand the known microbial diversity repertoire of loggerhead turtles, highlighting interesting taxa specific to individual body sites. This study provides a comprehensive view of the loggerhead sea turtle bacterial microbiota and marks the first report of distal gut fungal communities that contributes to establishing a baseline understanding of loggerhead sea turtle holobiont.

**Supplementary Information:**

The online version contains supplementary material available at 10.1007/s00248-024-02388-x.

## Introduction

Microbial communities associated with animal hosts play crucial roles in various aspects of the host’s development, physiology, immune response, metabolism, and reproduction, and may have an impact on the host’s evolutionary potential [1, 2]. While the importance of sea turtle epibiosis with macro-epibionts (> 1 mm) such as barnacles has long been recognized [3], research on the microbial epibionts and endobionts of sea turtles has only recently gained attention [4–6]. Sea turtles hold a unique ecological role as keystone species, connecting terrestrial and coastal habitats, but they are also highly vulnerable to anthropogenic threats, such as climate change, disruption of feeding and breeding habitats, egg poaching, and accidental bycatch [7–9]. To lessen some of the pressure sea turtles face today, global conservation efforts have focused on safeguarding female turtles and their nesting areas, together with the rehabilitation of injured and sick turtles [8]. Motivated by the aim of enhancing the rehabilitation and conservation of wild animals and their associated microbiomes, the studies of sea turtle gut, skin, egg, and nest microbiomes have become a forefront in sea turtle conservation research, building upon cultivation-based and pathogen-oriented research in the veterinary domain [10–12].

Loggerhead sea turtles (*Caretta caretta*) are a widely distributed species and are classified as vulnerable by the IUCN Red List of threatened species [13]. However, the Mediterranean subpopulation of loggerheads is categorized as “Least Concern” due to long-term successful conservation efforts [8, 13]. Currently, loggerhead sea turtles’ microbiota is the second most studied, preceded only by green turtles (*Chelonia mydas*) [6]. Previous studies that used culture-dependent approaches have identified the most common pathogens associated with mucosal surfaces, skin lesions (such as bacterial families *Aeromonadaceae*, *Pseudomonadaceae*, *Enterobacteriaceae*), and hatchling failure (*Fusarium* spp.), with the presence of antibiotic resistance genes indicating loggerheads as sentinels of antibiotic pollution in the Mediterranean [11, 14–22]. Recent investigations using next-generation sequencing approaches to study the loggerhead microbiota shed light on the bacterial community structure and composition of the gastrointestinal tract [5, 23, 24], the impact of rehabilitation on mucosal bacteriomes [24, 25], the effects of plastic pollution on the gut bacteria [26], variations in microbial communities driven by localities [27, 28] or turtle anatomy [28, 29], and host-microbial coevolution patterns [30]. On the other hand, the fungal communities associated with marine reptiles, including sea turtles, have received limited attention using cultivation-independent approaches, despite the vulnerability of sea turtles to infections caused by *Fusarium* spp. during early development [18]. Recent work by Guo et al. [31] provided initial insights into the fungal communities found on carapace (healthy and ulcerated), in faeces, and in the seawater of green turtle juveniles undergoing rehabilitation; however, comprehensive surveys of endobiotic fungal communities in loggerhead sea turtles have not yet been conducted. Given the ecological significance of loggerhead sea turtles in the Mediterranean basin ecosystem, their role as sentinels for pollution, and their potential to act as vectors for zoonotic diseases, a comprehensive approach including eukaryotic microorganisms is necessary to understand the loggerhead sea turtle microbiota. This knowledge will contribute to the advancement of current conservation practices and future microbial stewardship efforts [32].

The objective of this study was to investigate the bacterial and fungal communities associated with loggerhead sea turtles found in the Adriatic Sea. More specifically, we aimed to analyse the bacterial communities in the cloacal, oral, and enclosure samples, as well as fungal communities of cloacal and enclosure tank water samples using amplicon sequencing targeting the V3-V4 (V34) region of 16S rRNA gene and the ITS2 region of nuclear ribosomal genes, respectively. Furthermore, when available, we aimed to compare the bacterial communities of carapace biofilm samples corresponding to turtles in this study out of which some were previously analysed as a part of our earlier study on sea turtle epibiosis [28]. By combining these datasets, we provide a comprehensive overview of the environmental, surface, and internal microbiota of the loggerhead turtles, establishing a baseline for future holobiont approaches to studying the loggerhead sea turtles.

## Methods

### Loggerhead Sea Turtle Sampling

**Fig. 1:**
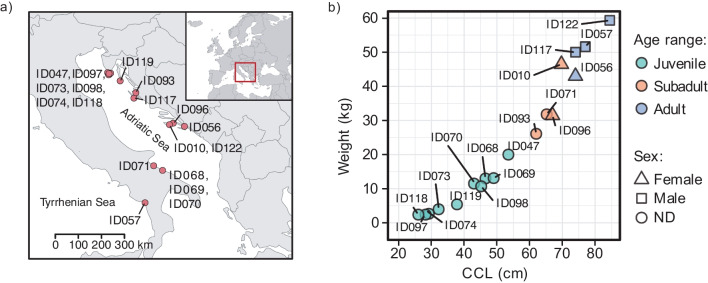
Locations and body measurements of loggerhead sea turtles with corresponding IDs. (**a**) Map of locations where loggerhead sea turtles were found prior to transport to rehabilitation centres and sampling. (**b**) Relationship of loggerheads’ weight in kilogrammes and curved carapace length in centimetres (CCL). The sex of each turtle is indicated by shape [female triangle, male square, and not determined (ND) circle], while age range was determined as follows: juveniles ≤ 59.9 cm, subadults 60–69.9 cm, adults ≥ 70 cm

Loggerhead sea turtles investigated in this study were found at various locations along the Adriatic Sea coast from 2019 to 2021 (Fig. 1a) and transported to two locations where the sampling was conducted: at the Sea Turtle Clinic (STC) of the Department of Veterinary Medicine of University of Bari “Aldo Moro” in Italy and the Sea Turtle Rescue Center Aquarium Pula in Croatia. The turtles were sampled immediately upon their arrival to the rehabilitation centres or during/after rehabilitation, and prior to release (Table S1). A total of 18 loggerhead sea turtles and 8 respective enclosures were included in the sampling. Turtles were classified as juveniles (*n* = 10), subadult (*n* = 4), and adults (*n* = 4) according to their size [33] and sex determination was based on observable physical characteristics when possible (Table 1; Fig. 1b). Additional information about sampling procedures and the loggerhead population surveyed in this study can be found in Supplementary Methods and Table S2.

**Table 1:** Information about loggerhead sea turtles and their corresponding endozoic (16S rRNA gene and fungal ITS2 region) and epizoic (16S rRNA gene) samples (V4 — Kanjer et al. [28], V34 — this study). The turtles were retrieved at various locations in the Adriatic Sea and were admitted to Sea Turtle Rescue Center Aquarium Pula (Croatia) unless indicated otherwise (^T^Tyrrhenian Sea; ^STC^The Sea Turtle Clinic at University of Bari, Italy). The abbreviations are as follows: *Clo*, cloaca; *Orl*, oral cavity; *TW*, tank water; *Car*, carapace; *NA*, not available; *ND*, not determined

**Turtle ID**	**Turtle name**	**Sampling event**	Endozoic 16S/ITS2 sample presence (+) or absence (-)^*^			**Epizoic 16S sample ID (region)**	**Sex**	**Age range**
			**Clo**	**Orl**	**TW**	**Car**		
ID010	Merry Fisher	0084	+/+	-/-	+/+	TB139 (V4)	Female	Subadult
ID047	Žal	0064	+/+	+/-	+/+	TB115 (V4)	ND	Juvenile
ID056	Samba	0073	+/+	+/-	+/+	TB117 (V4)	Female	Adult
ID057	Angelo^T, STC^	0074	+/+	+/-	-/-	NA	Male	Adult
		0092	+/+	+/-	-/-	TB119 (V4)		
ID068	Kanooh^STC^	0087	+/+	+/-	-/-	TB145 (V4)	ND	Juvenile
ID069	Kanfus^STC^	0088	+/+	+/-	-/-	NA	ND	Juvenile
ID070	Futon^STC^	0089	+/+	+/-	-/-	TB149 (V4)	ND	Juvenile
ID071	Cosmyn^STC^	0090	+/+	+/-	-/-	TB151 (V4)	ND	Subadult
ID073	Marvin	0093	+/+	+/-	+/+	TB155 (V4)	ND	Juvenile
ID074	Ryan	0094	+/+	+/-	-/-	TB157 (V4)	ND	Juvenile
ID093	Ella Ravka	0113	+/+	+/-	-/-	TB159 (V34)	ND	Subadult
		0119	+/+	+/-	+/+	NA		
ID096	Maro	0117	+/+	+/-	+/+	TB167 (V34)	Female	Subadult
		0118	+/+	+/-	+/+	NA		
ID097	Freewings	0120	+/+	+/-	+/+	NA	ND	Juvenile
ID098	Maksimus	0123	+/+	+/-	+/+	TB175 (V34)^**^	ND	Juvenile
ID117	Karlo Albano	0141	+/+	+/-	-/-	TB215 (V34)	Male	Adult
ID118	Oliver Raul	0142	+/+	+/-	-/-	TB217 (V34)	ND	Juvenile
ID119	Martin	0143	+/+	+/-	-/-	TB219 (V34)	ND	Juvenile
ID122	Luka Amadeo	0146	+/-	+/-	-/-	NA	Male	Adult
^*^Each sample ID has a 16S or ITS prefix, sampling event number, and suffix corresponding to sampling site: *C*, cloaca; *O*, oral cavity; *W*, tank water; for example, sample ID ITS0084C represents cloacal sample of fungal ITS2 sequences for sampling event 0084								
^**^Carapace sample was collected at a different sampling event (a month prior to endozoic sampling)								

The endozoic samples were collected from cloacal and oral cavities in triplicate by sterile synthetic swabs (Aptaca Nuova) as described in Filek et al. [25]. When available, enclosure tank water was collected in sterile containers, vacuum filtered on 0.2-μm sterile Whatman polycarbonate membrane filters (Sigma-Aldrich), and stored in 2-ml tubes in 96% EtOH. All samples were stored at −20 °C until DNA extraction and further processing. Additionally, corresponding epizoic carapace biofilm samples were obtained by randomly brushing the entire carapace using a toothbrush (Dentalux Classic, hard, Lidl) according to [28, 34] and the collected material was resuspended in 96% EtOH, and stored at −20 °C (Table 1). Each endozoic sequencing sample ID that is referred to in this manuscript has a 16S or ITS prefix, sampling event number, and suffix corresponding to sampling site: C — cloaca, O — oral cavity, W — tank water; for example, sample ID ITS0084C represents cloacal sample of fungal ITS2 sequences for sampling event 0084 (turtle ID010; Table 1). Epizoic samples have a TB prefix and numbering unrelated to sampling event.

### DNA Extraction and Sequencing

Total DNA from swabs and filters was extracted using the DNeasy PowerSoil kit (Qiagen) following the manufacturer’s instructions with several modifications: (1) the samples were incubated in C1 solution at 65 °C for 1 h; (2) instead of bead beating, PowerBead Tubes were vortexed horizontally for 10 min at maximum speed; and (3) all downstream incubation times at 2–8 °C were increased to 15 min.

The methods used for epizoic carapace biofilm samples that were sequenced for V4 region (ID010–ID074) were described by Kanjer et al. [28]. The DNA from biofilm scrapings (ID093–ID122) analysed only in this study was extracted via DNeasy PowerLyzer PowerSoil extraction kit (Qiagen) following the manufacturer’s instructions and modified as follows: (1) after addition of C1 solution, the samples were incubated at 70 °C for 10 min; (2) bead beating was performed at 30 Hz for 1 min in TissueLyzer Retsch Qiagen; (3) 50 µl of C6 solution was used for DNA elution and incubated for 5 min at room temperature prior to centrifugation. Nuclease-free water (W4502, Sigma-Aldrich) was used as the negative control for the DNA extraction step and was processed using the DNA extraction kit in parallel to all the samples. The quality and quantity of extracted DNA were evaluated by BioSpec-nano (Shimadzu).

The extracted DNA from both endozoic and epizoic samples was stored at −20 °C and sent for Illumina MiSeq v3 300 × 2 bp paired-end sequencing to Microsynth, Switzerland. Primers used for sequencing the V34 region of 16S rRNA gene were 341F and 805R [35], and primers for fungal ITS2 region of the nuclear ribosomal gene were ITS3 and ITS4 [36].

### Bioinformatics and Statistics

The obtained sequences had non-biological sequences trimmed by the sequencing facility and checked for quality with FastQC [37]. Sequencing data is available at EMBL ENA at accessions PRJEB62752 and PRJEB68298 for 16S rRNA gene, PRJEB62762 for ITS2 region sequences, and from Kanjer et al. [28] PRJEB51458. The sequences were imported and analysed in QIIME 2 (versions 2021.8 and 2023.2) [38].

Statistical analyses were performed within QIIME 2 environment and with R. Alpha diversity indices, including Shannon’s entropy, Pielou’s evenness, Faith’s phylogenetic diversity, and observed ASVs, were calculated via q2-diversity plugin. The Kruskal-Wallis rank-sum test was employed to determine differences between selected groups, followed by post hoc pairwise comparisons using the Wilcoxon rank-sum exact test. For beta diversity analyses, rarefied data (sequencing depth determined by alpha rarefaction curves) were explored using the q2-diversity plugin with Bray-Curtis, Jaccard, unweighted UniFrac, and weighted UniFrac distances [39]. Compositional data analysis on non-rarefied datasets was performed by calculating robust Aitchison distances using the q2-deicode plugin or the R package vegan v.2.6-4 [40–42]. Principal coordinate analysis (PCoA) was conducted on Bray-Curtis, Jaccard, and all UniFrac distances, while principal component analysis was performed for robust Aitchison (rPCA) using q2-diversity and q2-deicode, respectively. To assess the relative impact of factors (age range, sex, and duration of rehabilitation) on microbial communities, a multi-way permutational multivariate analysis of variance (Adonis2 PERMANOVA) with 9999 permutations was employed (Anderson, 2001) in R by using vegan v.2.6-4 and pairwise Adonis v.0.4.1 packages [43]. Resulting *p*-values from all pairwise tests were adjusted using the Benjamini-Hochberg false-discovery rate (FDR) correction for multiple comparisons (reported as *q*-values). Differential abundance analysis was used to identify differentially abundant (DA) features in sample site pairs by using the ANCOMBC package “ancombc2” function in R [44, 45]. Default parameters were used and pairwise testing was enabled (with Holm’s method for adjusting *p*-values, reported as q-values), except for DA testing in V4-trimmed sequences where the “struc_zero” was set to “TRUE” to exclude structural zeros based on sampling sites. Log fold change (LFC) indicates the scale of differential abundance between differentially abundant features. Features with *p*-value < 0.05 were reported as differentially abundant, and *q*-value < 0.05 as significantly differentially abundant.

Data exploration and visualizations were conducted by using R v.4.3.0 in RStudio (R Core Team 2023) with packages listed above and in Supplement and qiime2R v.0.99.6 [46], tidyverse v2.0.0 [47], ggplot2 v3.4.2 [48], Microsoft Excel, and Adobe Illustrator. Additional details of sample processing and data analyses are available in the Supplementary Methods.

## Results

### Endozoic and Tank Water Bacterial Communities

Altogether, 50 endozoic and water samples (plus one negative control) were sequenced, and 7,110,067 high-quality sequences were obtained (median frequency per sample was 107,522, min. 2, max. 911,443). Denoising yielded a total of 11,105 ASVs. After filtering mitochondrial and chloroplast sequences, the number of ASVs decreased to 10,946. Forty-three samples yielded enough high-quality reads (at minimum sequencing depth above 10,000) for downstream analyses (19/21 cloacal, 16/20 oral, and 8/9 tank water samples).

**Fig. 2:**
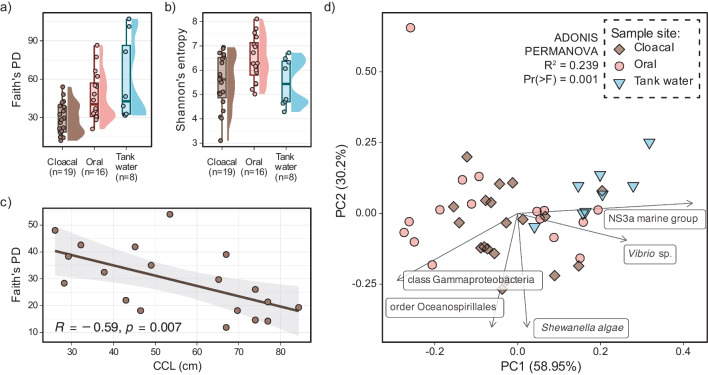
Bacterial community structure and diversity in loggerhead sea turtles’ cloacal, oral, and tank water samples. Alpha diversity boxplots with sample density for Faith’s phylogenetic diversity (**a**) and Shannon’s index (**b**) per sample site. (**c**) Pearson’s correlation between Faith’s phylogenetic diversity and curved carapace length (CCL). (**d**) Robust Aitchison PCA biplot with highly ranked features as loadings. Cloacal samples are depicted by diamonds, oral samples by circles, and tank water by inverted triangle shapes

The highest number of observed features (OF) and phylogenetic diversity (Faith’s PD) was found in tank water samples (median OF = 603, IQR = 450; median Faith’s PD = 42.8, IQR = 53.9), followed by oral samples (median OF 473, IQR = 428; median Faith’s PD 40.2, IQR = 26.2), and cloacal samples (median OF = 308, IQR = 235; median Faith’s PD = 28.4, IQR = 20) (Fig. 2a). ASV richness and evenness (Shannon’s index) were highest in oral samples (median = 6.28, IQR = 1.33), followed by cloacal (median = 5.62, IQR = 1.66) and tank water samples (median = 5.44, IQR = 1.67) (Fig. 2b). Alpha diversity showed significant differences among sample sites (Kruskal-Wallis rank-sum test) for OF and Faith’s PD (*p*-value < 0.01), while pairwise sample site comparisons (Wilcoxon rank sum test) showed differences between cloaca vs. oral samples and cloaca vs. tank water (*q*-value < 0.05). Shannon’s index showed weak statistical difference among samples sites (*p*-value = 0.04). Pearson’s correlation on alpha diversity indices against size of the turtle (CCL) in individual sample site groups showed strong negative correlation between cloacal samples and OF and Faith’s PD (*R* = −0.67, *p*-value = 0.002 and *R* = −0.59, *p*-value = 0.007, respectively) (Fig. 2c), while Shannon’s index showed weaker negative correlation with CCL (*R* = −0.53, *p*-value = 0.021). Oral and tank water alpha diversity did not show any correlation effects. Further, within cloacal samples, differences were detected (Kruskal-Wallis *p*-value < 0.05) in age range (OF, pairwise Wilcox: adult vs. juvenile *q*-value = 0.024; Faith’s PD, pairwise: adult vs. juvenile *q*-value = 0.024) and sex of the turtles (OF, pairwise Wilcox: male vs. ND *q*-value = 0.004; Faith’s PD, pairwise: male vs. ND *q*-value = 0.018), which are directly related to turtles’ size.

Bacterial communities among samples sites differed based on PERMANOVA for Bray-Curtis (*R*^2^ = 0.09, Pr(> *F*) = 0.001), Jaccard (*R*^2^ = 0.08, Pr(> *F*) = 0.001), unweighted and weighted UniFrac (*R*^2^ = 0.12, Pr(> *F*) = 0.001; *R*^2^ = 0.13, Pr(> *F*) = 0.001, respectively), and robust Aitchison distances (*R*^2^ = 0.24, Pr(> *F*) = 0.002). Pairwise PERMANOVA detected differences between all sample site pairs for most beta diversity metrics except robust Aitchison where only cloaca vs. tank water and tank water vs. oral samples pairs were significantly different (Pr(> *F*) ≤ 0.002). Highly ranked ASVs impacting the distribution of samples on rPCA biplot were assigned to Gammaproteobacteria, order Oceanospirillales, *Shewanella algae*, *Vibrio* sp., and NS3a marine group (Fig. 2d). Within cloacal samples, significant differences in bacterial communities were detected between adults vs. juveniles (Bray-Curtis, Jaccard, and unweighted UniFrac Pr(> *F*) < 0.05) and ND vs. males (all distances except robust Aitchison Pr(> *F*) < 0.05) or females (Jaccard and weighted UniFrac Pr(> *F*) < 0.05). In oral samples, there were differences between early vs. late hospitalization duration (all distances except robust Aitchison Pr(> *F*) < 0.05) and early vs. mid (weighted UniFrac Pr(> *F*) < 0.05) hospitalization durations.

Cloacal bacterial communities differed between age groups based on PERMANOVA for Bray-Curtis (*R*^2^ = 0.15, Pr(> *F*) = 0.018), Jaccard (*R*^2^ = 0.14, Pr(> *F*) = 0.008), and unweighted UniFrac distance (*R*^2^ = 0.16, Pr(> *F*) = 0.013). Pairwise PERMANOVA showed significant differences only between adults and juveniles for all above-mentioned diversity measures (Pr(> *F*) ≤ 0.009). The differences between adults and juveniles are observed in community composition and structure as well: phylum Verrucomicrobiota appears more often in juveniles, juveniles also have higher RA of Rhodobacterales (Alphaproteobacteria), Cardiobacterales (Gammaproteobacteria), Kineosporiales (Actinobacteriota), Oligoflexales (Bdellovibrionota), *Arcobacteraceae* (Campylobacterales), and more Flavobacteriales (Bacteroidota) than adults. On the other hand, adults carry more Bacteroidales (Bacteroidota), Pasteurellales (Gammaproteobacteria), *Helicobacteraceae* and *Campylobacteraceae* (Campylobacterales), and *Leptotrichiaceae* (Fusobacteriales). The low number of samples (≤ 10) in each age group prevented us from further differential abundance analyses. Detailed taxonomic composition of bacterial communities found in oral, cloacal, and tank water samples is reported in Supplementary Results, Table S3 with additional visualizations available at Github.

**Fig. 3:**
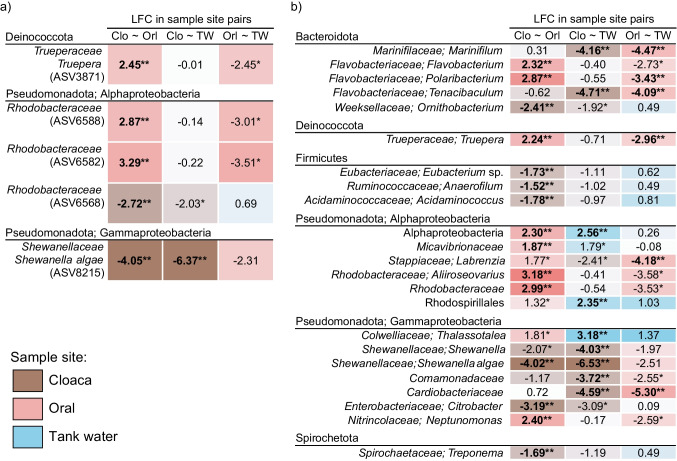
Differential abundance analysis by ANCOM-BC2 for ASVs (**a**) and taxa collapsed to species level (**b**) in cloacal, oral, and tank water samples. Only differentially abundant taxa in oral or cloacal sample sites are shown. Sample site abbreviations are “Clo” for cloaca; “Orl” for oral; and “TW” for tank water samples. The first sample site listed in sample site pairs was used as a denominator for log fold change (LFC) calculations in pairwise testing, thus the LFC value for that body site being < 0. Single asterisk (*) indicates significant differential abundance (*p*-value < 0.05), while double asterisk (**) indicates significance after adjusting for multiple testing (*q*-value < 0.05)

Differential abundance analysis detected 33 significantly DA features (post adjusting for multiple testing) out of which two were differentially abundant in cloaca (ASV467 *Rhodobacteraceae* and ASV4007 *Shewanella algae*), and three in oral samples (ASV9794 *Truepera* sp., ASV6166 and ASV467 both belonging to *Rhodobacteraceae*) (Fig. 3a). Tank water had 28 DA ASVs when tested against cloaca or oral samples, most of which belonged to typical marine taxa (*Cryomorphaceae*, NS3a marine group, SAR406 clade, *Rhodobacteraceae*, *Nitrincolaceae*, etc.). When collapsed to species level, 75 significantly DA taxa were detected, out of which 13 in cloaca and 15 in oral samples (Fig. 3b). Depending on the tested sample site pair, several features would be DA in both oral and cloacal samples when compared to tank water, e.g., genera *Marinifilum*, *Tenacibaculum*, and *Labrenzia*, members of *Cardiobacteriaceae* and *Comamonadaceae* families (Fig. 3b).

### Endozoic and Tank Water Fungal Communities

Overall, 29 samples (plus one negative control) were sequenced, and 2,208,729 high-quality sequences were obtained (median frequency per sample was 69,203, min. 11,722, max. 150,142). Denoising yielded a total of 9547 ASVs. Based on alpha rarefaction curves, 27 samples yielded enough high-quality reads (at minimum sequencing depth at 25,000 reads) for downstream statistical analyses requiring rarefied data (19/20 cloacal and 8/9 tank water samples). All samples except negative control were used in compositional data analyses.

**Fig. 4:**
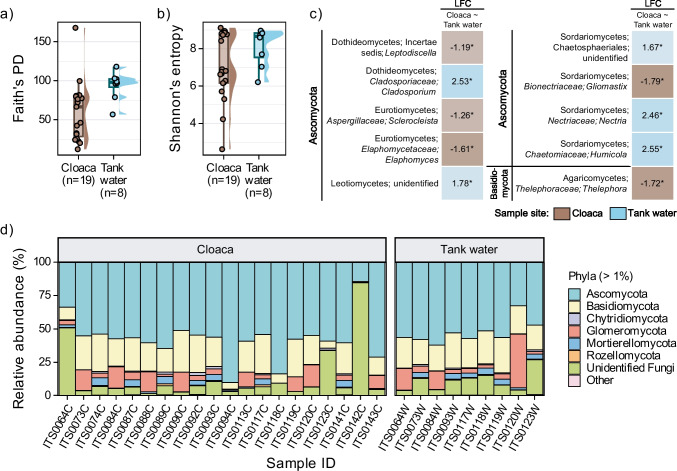
Structure and composition of fungal microbial communities in cloaca and tank water samples. Alpha diversity boxplots with sample density for Faith’s phylogenetic diversity (**a**) and Shannon’s index (**b**) per sample site. (**c**) Differential abundance analysis by ANCOM-BC2 for taxa collapsed to species level in cloacal and tank water samples. Cloacal sample site was used as a denominator for log fold change (LFC) calculations in pairwise testing, thus the cloacal LFC values being < 0. Single asterisk (*) indicates significant differential abundance (*p*-value < 0.05). (**d**) Relative abundance of fungal phyla in cloacal and tank water samples present above 1% in at least one sample. Sample IDs ending with “C” belong to cloacal samples, while sample IDs ending with “W” belong to tank water samples

Tank water samples had significantly higher number of observed features and phylogenetic diversity (median = 674, IQR = 97.8; Faith’s PD median = 98.0, IQR = 10.5) than cloacal samples (median = 509, IQR = 372, Faith’s PD median = 66.6, IQR = 51.7) (Fig. 4a) based on the Kruskal-Wallis rank-sum test (OF *p*-value = 0.008; Faith’s PD *p*-value = 0.007). Shannon’s diversity was not detected as significantly different between sample sites (tank water median = 8.66, IQR = 1.31; cloaca median = 6.76, IQR = 2.58) (Fig. 4b). There were no significant differences in alpha diversity values within cloacal or tank water fungal communities when tested for age range, sex, or rehabilitation duration. The only beta diversity metric that showed significant differences between tank water and cloaca was unweighted UniFrac (*R*^2^ = 0.06, Pr(> *F*) = 0.042). Unweighted UniFrac PCoA showed unclear sample groupings; however, there is a separation of cloacal and corresponding tank water samples on PCA1 axis (Fig. S1a, corresponding samples connected with dashed lines). Compositional data analysis rPCA did not show clear separation of samples sites, and top ranked ASVs belonged to *Preussia flanaganii*, genus *Tetracladium*, order *Xylariales*, genus *Rhizoctonia*, and family *Nectriaceae* (Fig. S1b). When collapsed to species level, cloacal and tank water samples each had five differentially abundant fungal taxa detected. In cloacal samples, DA taxa were *Leptodiscella*, *Elaphomyces*, *Sclerocleista*, *Gliomastix*, and *Thelephora*; and in tank water, they were *Cladosporium*, unidentified Leotiomycetes, unidentified Sordariomycetes (Chaetosphaerilaes), *Nectria*, and *Humicola* — although none of them was statistically significant after correction for multiple testing (Fig. 4c). Cloacal and tank water fungal communities were represented mostly by phyla Ascomycota (average RA ± standard deviation in cloaca and tank water = 57 ± 15% and 53 ± 9%, respectively), Basidiomycota (19 ± 9% and 22 ± 3%), Glomeromycota (7 ± 6% and 10 ± 12%), Mortierellomycota (2 ± 2% and 3 ± 2%), and unidentified Fungi (13 ± 20% and 11 ± 7%) (Fig. 4d). Detailed taxonomic composition of fungal communities found in cloacal and tank water samples is reported in Supplementary Results and Table S4.

### Epizoic and Endozoic Bacterial Communities

After trimming epizoic, endozoic, and tank water sequences to V4 region of 16S rRNA gene (65 samples in total) and denoising, 10,072,866 high-quality sequences were merged across all sequencing events (median frequency per sample was 126,629, min. 0, max. 946,183) and yielded 13,263 ASVs. Due to reduced resolution after trimming the longer V34 region to shorter V4 region, the number of ASVs for cloacal, oral, and tank water samples was expectedly lower (7725 in V4 vs. 11,105 in V34 sequences).

**Fig. 5:**
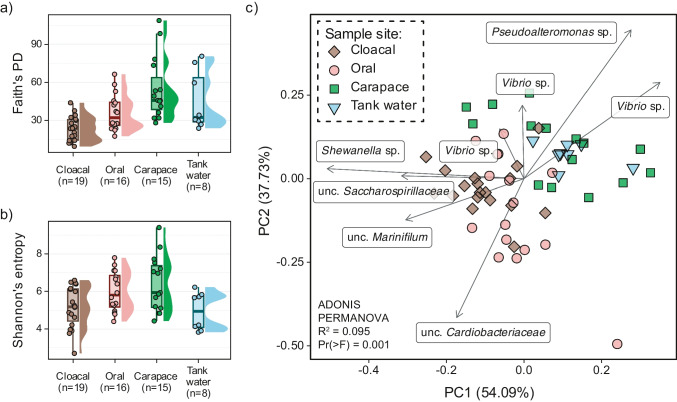
Bacterial community structure and diversity in loggerhead sea turtles’ carapace, cloacal, oral, and tank water samples.  Alpha diversity boxplots with sample density for Faith’s phylogenetic diversity (**a**) and Shannon’s index (**b**) per sample site. (**c**) Robust Aitchison PCA biplot with highly ranked features as loadings. Cloacal samples are depicted by diamonds, oral samples by circles, carapace samples by squares, and tank water by inverted triangle shapes

Carapace samples had the highest median species richness and diversity (OF median 732, IQR = 420, Faith’s PD median = 45.7, IQR = 25.2; median Shannon’s index = 5.94, IQR = 2.22), which was followed by tank water, oral, and cloacal samples (Fig. 5a and b). The differences between sample sites’ alpha diversity were detected as statistically significant (Kruskal-Wallis rank-sum test); however, pairwise comparisons showed differences only in Faith’s phylogenetic diversity and OF: between cloaca and all other sample sites (except tank water based on OF), oral cavity and carapace. All beta diversity metrics showed significant differences between body sites for Bray-Curtis (*R*^2^ = 0.13, Pr(> *F*) = 0.001), Jaccard (*R*^2^ = 0.10, Pr(> *F*) = 0.001), unweighted and weighted UniFrac (*R*^2^ = 0.16, Pr(> *F*) = 0.001; *R*^2^ = 0.18, Pr(> *F*) = 0.001, respectively), and robust Aitchison distances (*R*^2^ = 0.09, Pr(> *F*) = 0.001). Pairwise PERMANOVA detected differences between all sample site pairs (Pr(> *F*) < 0.01). Highly ranked ASVs impacting the distribution of samples on rPCA biplot belonged to *Pseudoalteromonas* sp., *Vibrio* sp., *Shewanella* sp., uncultured *Saccharospirillaceae*, uncultured *Marinifilum*, and uncultured *Cardiobacteriaceae* (Fig. 5c). The taxonomic composition of endozoic samples and tank water using the V4 sequences resembled the one detected with V34 and it is reported in Table S5. Additional information on taxonomic composition of epizoic bacterial communities in carapace samples is reported in Supplementary Results.

**Fig. 6:**
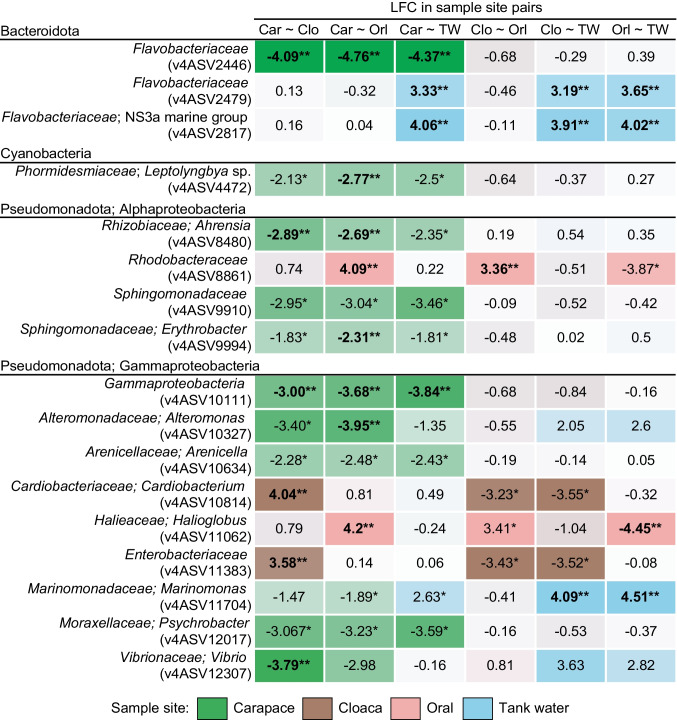
Differential abundance analysis by ANCOM-BC2 (with structural zeros excluded) for ASVs in carapace, cloacal, oral, and tank water samples. Sample site abbreviations are “Car” for carapace; “Clo” for cloaca; “Orl” for oral; and “TW” for tank water samples. The first sample site listed in sample site pairs was used as a denominator for log fold change (LFC) calculations in pairwise testing, thus the LFC value for that body site being < 0. Single asterisk (*) indicates differential abundance (*p*-value < 0.05), while double asterisk (**) indicates significance after adjusting for multiple testing (*q*-value < 0.05)

Differential abundance analysis on ASVs (structural zeros excluded) detected 17 significantly DA ASVs across all sample sites (Fig. 6). A member of *Rhodobacteraceae* family (v4ASV8861) and *Halioglobus* sp. (v4ASV11062) were consistently DA in oral samples while the same goes for *Cardiobacterium* sp. (v4ASV10814) and a member of *Enterobacteriaceae* family (v4ASV11383) in cloaca. In carapace samples, a member of *Flavobacteriaceae* family (v4ASV2446), cyanobacteria *Leptolyngbya* sp. (v4ASV4472), *Ahrensia* sp. (v4ASV8480), *Sphingomonadaceae* (v4ASV9910), *Erythrobacter* sp. (v4ASV9994), *Gammaproteobacteria* (v4ASV10111), *Alteromonas* sp. (v4ASV10327), *Arenicella* sp. (v4ASV10634), *Psychrobacter* sp. (v4ASV12017), and *Vibrio* sp. (v4ASV12307) were DA relative to other sample sites, while *Marinomonas* sp. (v4ASV11704) was DA abundant in carapace tested against endozoic samples, but not tank water. In tank water, a member of *Flavobacteriaceae* (v4ASV2446) and NS3a marine group (v4ASV2817) were consistently DA (Fig. 6). Differential abundance analysis on taxa collapsed to species level is reported in Supplementary Results and Fig. S2.

## Discussion

This study provides an overview of the bacterial and fungal communities inhabiting oral and cloacal environments of loggerhead sea turtles. Additionally, we aimed to investigate bacterial communities in carapace samples in relation to the gastrointestinal tract and tank water to explore possible connections between two habitats. The carapace exhibited the highest bacterial diversity, followed by oral samples, influenced by the tank water environment, and then cloacal samples. Each sampling site had distinct microbial communities and cloacal bacterial diversity negatively correlated with turtle size and age. Conversely, fungal communities in the cloaca were distinct from tank water and showed high heterogeneity among individual turtles, with no discernible patterns related to age or sex.

### Cloacal Bacterial Diversity and Structure Changes with the Turtle Age

Similarly to previous studies on cloacal microbiota in Adriatic loggerhead sea turtles, we observed changes in bacterial richness, diversity, and structure, negatively correlating with loggerheads’ CCL and, consequently, their age [24]. According to research on vertebrate gut microbiomes, it is expected that the bacterial diversity increases with body size in animals with complex digestive systems, like ruminants, and decreases in animals with simple guts, often omnivores or carnivores [49]. Loggerhead sea turtles are omnivores and exhibit an ontogenetic shift from oceanic-pelagic habitats as juveniles to neritic-benthic feeding grounds as adults. However, in the Mediterranean Sea, they employ an “amphi-habitat strategy”, where juveniles, subadults, and adults share feeding grounds and similar feeding behaviours [50, 51]. Differences in diet, jaw size, bite force, and diving ability between juveniles and adults may result in distinct diets and digestive physiologies, potentially reflected in cloacal bacterial communities as increased richness. Studies on loggerhead diet show no significant differences in stomach contents among adults, subadults, and juveniles, but it is worth noting that softer prey like tunicates or jellyfish may not be as detectable due to easier digestion and lack of hard remains in morphology-based studies [50].

In this study, juveniles exhibited higher abundance and more frequent presence of *Flavobacteriaceae* and *Tenacibaculum* spp. in oral and cloacal samples than subadult and adult turtles. *Tenacibaculum* spp. are marine pathogens possibly carried by cnidarians and ctenophores as vectors [52, 53], suggesting a higher proportion of soft prey in juveniles’ diet. This study was conducted during non-winter months when jellyfish populations increase due to higher water temperatures potentially influencing prey availability. Adults likely have access to these prey types but also consume more challenging prey inaccessible to juveniles. Notably, in other studies, *Tenacibaculum* spp. were highly abundant on loggerhead skin as well [28], indicating their propensity to inhabit marine vertebrate surfaces and gastrointestinal tracts, irrespective of animal’s diet. While in previous research the *Mogibacteraceae* family detected in faeces correlated with CCL [24], we did not detect it in our data, possibly due to different taxonomy databases being utilized (Greengenes vs. SILVA in our study). BLAST analysis showed ASVs assigned as Peptostreptococcales-Tissieralles closely related to *Mogibacterium kristiansenii* but it still did not exhibit a CCL correlation.

### Loggerhead Cloacal Communities Are a Promising Source of Novel Campilobacterota

Members of the Campilobacterota phyla, particularly *Arcobacteriaceae*, *Campylobacteraceae*, and *Helicobacteraceae*, have recently been found to form unique, cold-adapted communities in ectothermic reptiles [54]. As expected, and based on their physiology and lifestyle, the *Arcobacter* genus, which can thrive at atmospheric oxygen levels and prefers lower temperatures, was present in all sample sites. However, it was more abundant in oral than in cloacal samples and was rarely detected in tank water. The *Helicobacter* and *Campylobacter* genera, which are vertebrate-associated, were observed in cloacal and occasionally oral samples. Notably, one adult male (ID057) showed a higher prevalence of *Helicobacter*, reaching up to 20% relative abundance in the cloaca, represented by a single ASV assigned as *Helicobacter* sp. and not found in any other sample. This suggests a potential overgrowth or infection despite the relatively good clinical status of the individual observed at the time. Therefore, like other reptiles, loggerhead sea turtles could serve as a valuable source of previously undiscovered cold-adapted *Campylobacter* and *Helicobacter* species, as well as mucosal *Arcobacter* species.

### Oral Bacterial Communities Harbour Distinct Taxa and Could Reflect Recent Diet

In oral samples, the genera *Truepera* and *Halioglobus* showed differential abundance. *Trueperaceae*, a relatively new family, includes one isolate *Truepera radiovictrix*, known for radiation resistance and thriving in extreme conditions like other Deinococci members [55]. *Truepera* was also a dominant part of the oral microbiota in splendid japalure lizards [56], but its role and functions in reptile oral microbiomes remain unclear. Tolerance to extreme environments and ability to use diverse carbon sources may allow *Truepera* spp. to outcompete other taxa on reptilian oral mucosa. The genus *Halioglobus* is typically found in seawater and marine sediments, and has been associated with dinoflagellate blooms and starving, green-lipped mussels [57, 58]. We have previously reported *Halioglobus* in oral samples of loggerhead sea turtles [25]. *Halioglobus* bacteria likely derive from loggerhead prey such as mussels or oysters rather than being an intrinsic property of sea turtle mucosal surfaces, given the limited information on host-associated *Halioglobus*. Similarly, the high relative abundance of the genus *Exiguobacterium* in oral and cloacal samples of one juvenile loggerhead (ID069) sampled upon admission may indicate recent feeding, as some *Exiguobacterium* species are commonly found on shrimp or algae [59–61].

### Carapace Microbiota Is Distinct, yet Can Harbour Taxa Specific to Oral or Cloacal Microbiota

The carapace and skin of loggerhead sea turtles harbour diverse microbial biofilms, rich in prokaryotes, microeukaryotes, and macroeukaryotes like barnacles and algae [3, 28]. These surface microbial communities vary with the turtle’s geographical location, carapace condition, and the sampled anatomical site [28, 29]. They can be considered as microbial reservoirs and “diversity hotspots” in otherwise scarce environments [62, 63]. In our study, we found expectedly distinct microbial communities in the carapace, oral mucosa, and cloaca, although many microbial taxa were present across all body sites, including potential zoonotic pathogens primarily from the cloaca. Oral and cloacal samples shared several differentially abundant microbial taxa (*Tenacibaculum*, *Cardiobacteraceae*, *Campylobacter*), suggesting co-inhabitation of the gastrointestinal tract. The carapace microbiota differed significantly from tank water, with indication of transfer of certain taxa from carapaces to tank water or vice versa. Taxa like *Alteromonadaceae* and *Colwelliaceae* (*Thalassotalea*), differentially abundant on carapaces in this study, were prominent in enclosure tank water in prior studies that did not examine carapace bacterial communities [24], possibly originating from captive turtles’ carapaces. The implications of the interplay between microbes from different body sites on loggerhead sea turtles in their natural habitats and during captivity are not yet clear. However, it is essential to consider surface microbiota and potential opportunistic pathogens, especially when rehabilitating severely injured and possibly immunocompromised individuals.

### Fungal Communities of Loggerhead Cloaca Are Highly Heterogeneous and Diverse

Fungal communities in loggerhead sea turtles’ cloaca and tank water exhibit high variability among individuals, with greater diversity observed in tank water. In this study, we could not attribute differences in the composition and structure of cloacal mycobiota to the turtles’ age, sex, or hospitalization status, possibly due to the limited sample size per sampling site and condition assessed. Conversely, captive juvenile green turtles displayed more consistent mycobiota richness and diversity across various sampling sites and health conditions, unaffected by environmental fungi [31]. The taxonomic composition of cloacal and tank water fungal phyla in our study aligns with previously reported marine fungi groups found in green sea turtle faeces [31], as well as marine algicolous fungi, sediments, and sponges [64]. Due to the lower resolution of the ITS2 gene marker and many unassigned fungal ASVs beyond the family level, this study offers just a general overview and serves as a foundation for further exploration of specific groups of interest.

We sporadically detected pathogenic fungal genera [18], with nine pathogenic genera found in both cloaca and tank water. Among these, *Fusarium* species (family *Nectriaceae*) are recognized sea turtle pathogens linked to reduced hatchling success [65]. Our study identified *Fusarium oxysporum* (a known pathogen), *Fusarium neocosmosporellium*, and *Fusarium waltergamsii*, not previously reported as sea turtle pathogens but related to known pathogenic species in the *Fusarium solani* species complex [18, 66]. *Fusarium* ASVs were relatively less abundant, except in one sample where they reached 8%. However, some ASVs assigned to the *Nectriaceae* genus may belong to *Fusarium* species as ITS region can be insufficient in detecting *Fusarium* species [67], potentially affecting our reported abundance of pathogenic fungi in cloacal and tank water samples.

The origin of these fungi — whether they are metabolically active and intrinsic to the sampled turtle population or introduced from the environment as spores or through food — is unclear. The impact of these fungi on the host, the interaction with the turtle’s immune system and physiology, and their potential host association remain unknown. Many fungal ASVs detected in this study belong to primarily terrestrial taxa, suggesting possible terrestrial sources during rehabilitation or a lack of marine fungal sequences in taxonomy databases. Additionally, numerous reads were assigned only as “Fungi”, indicating either undiscovered fungal taxa, lack of representative sequences, or possible turtle host origin.

### Low Biomass of Samples Is a Potential Limitation to Interpreting Results

Microbial composition results should be interpreted with caution due to low biomass collected with swabs and fewer fungal cells compared to bacterial cells in vertebrate guts [68]. Negative control (sterile water) sequenced with bacterial primers showed a small number of reads that were also detected in low-read samples, indicating potential contamination during DNA extraction and sequencing specifically for samples with low biomass (e.g. kitome) [69, 70]. Surprisingly, the negative control sequenced with fungal primers had significantly more reads than the bacterial negative control, containing numerous ASVs also found in cloacal and tank water samples. Although the community structures of cloacal and tank water samples differed from the negative control, indicating genuine fungal communities rather than random contaminants, the high number of reads from seemingly low-biomass samples or the “empty” negative control raises concerns about our sample handling and sequencing approach. To our knowledge, no studies addressed fungal contamination in DNA extraction kits as they do for prokaryotes, making it challenging to pinpoint the exact source of this fungal DNA. For future studies, we suggest including additional negative control samples at various sampling and processing stages when investigating undescribed gut-associated fungal communities.

## Conclusion

Loggerhead sea turtle-associated microbial communities are crucial for understanding the biology of these endangered reptiles and supporting conservation. In this study, we examined bacterial and fungal communities in juvenile, subadult, and adult loggerhead sea turtles. Our findings revealed distinct microbial communities in the carapace, cloaca, and oral mucosa, with characteristic taxa for each site and shared taxa between cloacal and oral samples (e.g. *Tenacibaculum*, *Moraxellaceae*, *Cardiobacteriaceae*, and *Campylobacter*). Cloacal bacterial communities exhibited decreasing diversity and changing composition with turtle age, likely due to shifts in diet as juveniles develop stronger bite force and diving capabilities. Microbial exchange with the environment appears to occur, particularly from turtles to tank water, especially from the carapace. Fungal communities in cloaca and tank water displayed high heterogeneity across individuals, with no age or clinical patterns, possibly due to limited samples and low fungal biomass in cloacal samples. Loggerhead sea turtles host complex microbial communities, including potential bacterial and fungal pathogens, which pose risks to handlers or the general public as changing turtle behaviour leads to increased human-turtle interactions. Despite growing research on loggerhead microbiomes, defining a healthy microbiome beyond bacteria remains challenging. Future research should prioritize establishing a description of healthy loggerhead microbiomes at different developmental stages (from eggs and hatchlings to juveniles and adults) to enhance conservation practices and explore potential probiotics and prebiotics for addressing their current and future needs.

### Electronic Supplementary Material

Below is the link to the electronic supplementary material.

Supplementary Material 1

Supplementary Material 2

Supplementary Material 3

Supplementary Material 4

Supplementary Material 5

Supplementary Material 6

Supplementary Material 7

Supplementary Material 8

## Data Availability

Sequencing data with non-biological sequences removed is available at EMBL ENA at accessions PRJEB62752 and PRJEB68298 for 16S rRNA gene and PRJEB62762 for ITS2 region sequences. Epizoic samples’ sequencing data obtained from Kanjer et al. [28] and used in this study can be found at ENA under accession PRJEB51458. Complete code and instructions for processing of the sequencing data and subsequent statistical analyses, results, and data visualizations are available at Github (https://github.com/kl-fil/2023-Filek_et_al._TBIOME_project) and ZENODO data depository (10.5281/zenodo.8054926).
