# Correction to: Loggerhead Sea Turtles as Hosts of Diverse Bacterial and Fungal Communities

**DOI:** 10.1007/s00248-024-02405-z

**Published:** 2024-06-26

**Authors:** Klara Filek, Borna Branimir Vuković, Marta Žižek, Antonio DiBello, Lucija Kanjer, Adriana Trotta, Marialaura Corrente, Sunčica Bosak

**Affiliations:** 1 Department of Biology, Faculty of Science, University of Zagreb, Horvatovac 102a, HR-10000 Zagreb, Croatia; 2 Ruđer Bošković Institute, Bijenička 54, HR-10000 Zagreb, Croatia; 3 Campus Universitario, University of Bari “Aldo Moro”, Via Orabona 4, 70125 Bari, BA Italy; 4 Department of Veterinary Medicine, University of Bari “Aldo Moro”, Str. Prov. Per Casamassima Km 3, 70010 Valenzano, BA Italy; 5 Diagnostic and Research Institute of Hygiene, Microbiology and Environmental Medicine, Medical University of Graz, Neue Stiftingtalstraße 6, 8010 Graz, Austria

**Correction to: Microbial Ecology**

10.1007/s00248-024-02388-x

The EMBL ENA Accession number for one of our datasets is wrongly listed in the published article. It should be listed as PRJEB68298 in Material and Methods and Data Availability Sections.

The corrected parts of the manuscript are:

**Bioinformatics and Statistics**

Sequencing data is available at EMBL ENA at accessions PRJEB62752 and PRJEB68298 for 16S rRNA gene, PRJEB62762 for ITS2 region sequences, and from Kanjer et al. [28] PRJEB51458.

**Data Availability**

Sequencing data with non-biological sequences removed is available at EMBL ENA at accessions PRJEB62752 and PRJEB68298 for 16S rRNA gene and PRJEB62762 for ITS2 region sequences.

