# Supplementary material for: Loggerhead Sea Turtles as Hosts of Diverse Bacterial and Fungal Communities
Source: Microb Ecol. 2024 May 30;87:79. doi: 10.1007/s00248-024-02388-x (PMC11139726; doi:10.1007/s00248-024-02388-x)
Supplement: Supplementary file 1 [file 248_2024_2388_MOESM1_ESM.pdf]

SUPPLEMENT

Loggerhead Sea Turtles as Hosts of Diverse Bacterial and Fungal Communities

Klara Filek, Borna Branimir Vuković, Marta Žižek, Lucija Kanjer, Adriana Trotta, Antonio Di Bello, Marialaura Corrente, Sunčica Bosak

SUPPLEMENTARY METHODS

Loggerhead sea turtle sampling

Loggerhead sea turtles were sampled from 2019 to 2021 in spring, summer, and autumn season (Table S1). The turtles were found strained, injured, or were unintentionally caught by fishermen in the Adriatic Sea area with one turtle named Angelo found in Tyrrhenian Sea, and transported to recovery centres in Bari, Italy (Sea Turtle Clinic DVM University of Bari) and Pula (Aquarium Pula) Croatia where they received appropriate treatment. Upon arrival, all turtles underwent examination for injuries and relevant information on their clinical status was collected (Table S2). Most turtles were found floating or stranded, exhibiting visible injuries caused by boat propellers or entanglement in fishing nets. Some turtles were in good clinical condition but unable to dive. Sampling was conducted by trained personnel in accordance with the 1975 Declaration of Helsinki, as revised in 2013 and the applicable national laws immediately upon the turtles' arrival to the rehabilitation centres or during/after rehabilitation, and prior to release (Table S1). Rehabilitation/ hospitalisation times were categorized in three groups: "early" (0-2 days), "mid" (3-10 days), and "late" (>10 days) (Table S1). The turtles were kept in individual enclosures with artificial saltwater at STC or recirculating and filtered local seawater in Aquarium Pula [1]. A total of 18 loggerhead sea turtles and 8 respective enclosures were included in the sampling, with three turtles sampled twice during their rehabilitation period: ID057 during the early stage, ID093 during both early and late stages, and ID096 in the late stage (Table S1). Turtle age range categories were established based on size measurements (curved carapace length) grouped according to [2] as follows: juveniles ≤59.9 cm, subadults 60-69.9 cm, adults ≥70 cm. Out of the turtles sampled, ten turtles were classified as juveniles, four as subadult (including two females), and four as adults (one female and three males).

Juvenile turtles had a median weight of 8 kg [interquartile range (IQR)=9.6] and a median CCL of 40.4 cm (IQR=16.2). Subadult turtles had a median weight of 31.6 kg (IQR=5.3) and median CCL of 66.1 cm (IQR=3.3), adult turtles had a median weight of 50.8 kg (IQR=5.3) and median CCL of 75.5 cm (IQR=4.9) (Table S1).

DNA extraction, sequencing and bioinformatics

Total DNA from all swabs and filters was extracted using the DNeasy PowerSoil kit (Qiagen) and from epizoic carapace samples using DNeasy PowerLyzer PowerSoil extraction kit (Qiagen). The samples were processed following the manufacturer's instructions with several modifications listed in the main Methods section. Nuclease-free water (W4502 Sigma-Aldrich) was used as the negative control for the DNA extraction step and was processed using the DNA extraction kits in parallel to

cloacal, oral and tank water samples. The swabs were transferred to PowerBead tubes with added two 3 mm sterile glass beads. The tank water samples' filters were first cut in half and then added to the tubes as described above. The remaining ethanol in the sample tubes was centrifuged at the highest speed for 5 minutes and the resulting pellet was then transferred to the respective PowerBead tube. The scrapings of epizoic carapace biofilms (ID093 – ID022) were firstly pelleted by centrifugation in conical 15 ml tubes at 1400 rfc for 10 minutes after which the ethanol supernatant was removed and 250 mg of material was used for the extraction.

The extracted DNA from both endozoic and epizoic samples was sequenced using services provided by Microsynth, Switzerland. Equal aliquots of extracted DNA reaching a minimum concentration of 5 ng/μL were sent as a template for 16S rRNA gene V34 region and ITS2 region of fungal nuclear ribosomal gene sequencing for bacteria and fungi, respectively.

The sequencing data were imported and analysed in QIIME 2 [3]. For ITS2 region the QIIME 2 version 2021.8 was used, and for 16S rRNA gene sequences it was version 2023.2, with the same plugins used for initial processing of the data albeit in versions corresponding to QIIME 2 version used. Each sequencing dataset was processed individually due to intrinsic properties of distinct sequencing events and gene markers used. The code for analyses and intermediate files pertaining to each sequencing event are available at GitHub (https://github.com/kl-fil/2023-Filek_et_al._TBIOME_project) and ZENODO data depository (doi:10.5281/zenodo.8054926).

The obtained sequences were demultiplexed by q2-demux and denoised by q2-dada2, producing amplicon sequence variants (ASVs, 100% operating taxonomic units). Sequences were then aligned by MAFFT [4] and FastTree2 in q2-phylogeny to construct a phylogenetic tree [5]. Taxonomy was assigned to ASVs through q2-feature-classifier [6, 7] classify-sklearn naive Bayes taxonomy classifier. For 16S rRNA gene sequences we used SILVA v. 138 [8] and for ITS2 reads we used UNITE v. 8.3 dynamic classifier [9]. Reads identified as chloroplasts and mitochondria were removed from the 16S rRNA gene sequencing data before further processing and statistical analyses. Negative controls and samples below 10 000 reads were excluded from all further statistical analyses. Additionally, to compare endozoic and epizoic 16S rRNA gene sequences, the sequences resulting from individual sequencing events were first trimmed to V4 region by cutadapt v.2023.2.0 using primers 515F and 806R [10, 11] followed by denoising. After denoising, the V4-trimmed epizoic, endozoic and tank water sequences were merged and analysed together.

SUPPLEMENTARY RESULTS

Taxonomic composition of bacterial communities in oral, cloacal and tank water samples

The bacterial communities of all samples were primarily represented [average relative abundance (RA) > 1% across sample site groups] by phyla Proteobacteria and Bacteroidota, Firmicutes and Campilobacterota in cloacal and oral samples, Verrucomicrobiota in cloacal and tank water samples, Bdellovibrionota in oral and tank water samples, Fusobacteriota and Spirochaetota in cloaca, Actinobacteriota in oral cavity, and Patescibacteria in tank water (Table S3). Proteobacteria consisted of 17 families (and corresponding taxa) with average RA above 1% in at least one sample site group: *Cardiobacteriaceae* (*Cardiobacterium* spp., *Suttonella* spp., and uncultured representatives),

Shewanellaceae (*Shewanella algae*), *Rhodobacteraceae* (unclassified), *Moraxellaceae* (*Acinetobacter* spp., *Psychrobacter* spp.), *Vibrionaceae* (*Vibrio* spp., *Vibrio fluvialis* at 30% RA in 16S0074O), *Pasteurellaceae* (unclassified at 24% RA in 16S0074C), *Neisseriaceae* (unclassified at 2-10% RA in 16S0074C, 16S0092C, 16S0141C, 16S0117O; *Stenoxybacter* spp. at ~7% RA in 16S0087C, 16S0089C, 16S0089O), *Pseudoalteromonadaceae* (*Pseudoalteromonas* spp.), *Saccharospirillaceae* (*Littoribacillus* spp., *Oceanobacter* spp., and uncultured), *Spongiibacteraceae*, *Sedimenticolaceae*, *Alteromonadaceae* (*Glaciecola* spp. in tank water), *Pseudomonadaceae* (*Pseudomonas* at 8% RA in 16S0088O), *Colwelliaceae* (*Colwellia* spp.), *Nitrincolaceae* (*Marinobacterium* spp. and *M. marisflavi*), *Oleiphilaceae*, and *Marinomonadaceae* (uncultured *Marinomonas* spp.). Bacteroidota consisted of seven families (> 1% average RA): *Flavobacteriaceae* (unclassified, *Flavirhabdus* spp., *Tenacibaculum* spp.), *Cryomorphaceae* (uncultured), *Saprospiraceae* (*Phaeodactylibacter* spp.), NS9 marine group (uncultured *Flavobacterium*), *Weeksellaceae* (*Moheibacter* sp.), Bacteroidaceae (*Bacteroides* spp.), and *Marinifilaceae* (uncultured). Firmicutes consisted of two families: *Lachnospiraceae,* and *Exiguobacteraceae* at 33% RA in 16S0088O. Campilobacterota consisted of three families: *Arcobacteraceae*, *Campylobacteraceae* (*Campylobacter* spp.), *Helicobacteraceae* (*Helicobacter* sp. at 19% RA in 16S0092C). Fusobacteriota consisted of two families: *Fusobacteriaceae* and *Leptotrichiaceae*, and Spirochaetota had the *Leptospiraceae* family with average RA above 1%. Verrucomicrobiota had no families above 1% average RA but in 16S0087C *Akkermansiaceae* were present at 8% RA. *Bdellovibrionota*, *Actinobacteriota*, and *Patescibacteria* had no families above 1% average RA across at least one sample site group.

Out of pathogenic bacteria reported in sea turtles as reviewed in [12], we detected *Cardiobacterium* spp. and *Suttonella* spp. (*Cardiobacteriaceae*) in cloacal and oral samples, *Vibrio* spp. mostly in oral and tank water samples, *Citrobacter* spp. (*Enterobacteriaceae*) at 2-5% RA in several cloacal and one oral sample, and *Morganella* spp. (*Morganellaceae*) at 12% RA in one cloacal sample (16S0073C). Other pathogenic bacteria were detected, albeit sparsely and at low RA (rarely reaching 1-3% RA in individual samples) (Table S3).

Our negative control sample analysed parallelly to other samples yielded 478 reads (23 ASVs) out of which 46% belonged to genus *Cupriavidus* (ASV8329). The rest of the reads were assigned to *Phyllobacterium* (9%, ASV6495), *Sphingomonas* (5%, ASV7577), Archaea *Nitrosopumilaceae* (4%, ASV14), *Lactobacillus* (3%, ASV4269), *Lachnoclostridium* (3%, ASV4451), *Pseudomonas* (3%, ASV10106), Archaea ANEM 2a-2b (3%, ASV26), *Caulobacteraceae* family (3%, ASV6031), *Bradyrhizobium* (2%, ASV6541), *Methylobacterium jeotgali* (2%, ASV6409), and Archaea *Nitrosopumilaceae* family (ASV18), *Paracoccus solventivorans* (ASV6950), *Stenotrophomonas* (ASV10578), *Blastocatellaceae* JGI 0001001-H03 metagenome (ASV131), *Lactobacillus iners* (ASV4273), Archaea order Methanomassiliicoccales (ASV62), *Rikenellaceae* RC9 gut group (ASV713), family *Microscillaceae* (ASV1470), family *Chitinophagaceae* (ASV778), *Sphingorhabdus* (ASV7595), *Cloacibacterium* (ASV2845), *Rhodomicrobium* (ASV6512) (all below 1%). Fourteen ASVs from negative control sample were detected in other samples as well. Low read samples (up to 5000 reads and all excluded from analyses) had 45% of total reads assigned to negative control *Cupriaviudus* ASV in comparison to high read samples that had 0.007%, followed by *Caulobacteraceae* and *Phyllobacterium*

at 7% and 3%, respectively (compared to high read samples that had <0.0001% reads assigned to those ASVs). The rest of negative control ASVs followed a similar pattern where they were comprising a substantial proportion of reads in low read vs. high read samples (Table S3). Top 3 ASVs in negative control (*Cupriavidus*, *Phyllobacterium*, *Sphinogomonas*) comprised 61% of total reads in negative control and 53% of total reads in low read samples (only 0.008% of total reads in high read samples).

Taxonomic composition of fungal communities in cloacal and tank water samples

In the fungal communities, within Ascomycota, most classes were found in cloaca and/or tank water above 1% average relative abundances (RA): Sordariomycetes (family *Nectriaceae* in cloaca and tank water, order Xylariales in cloaca), Pezizomycotina cls Incertae sedis (Pezizomycotina fam Incertae sedis), Leotiomycetes (family *Helotiaceae* and unclassified members of order Helotiales), Dothideomycetes (families *Phaeosphaeriaceae* in cloaca, *Sporormiaceae* in both, and *Lophiostomataceae* in tank water), Pezizomycetes (family *Pyronemataceae*), Eurotiomycetes, Saccharomycetes (cloaca). In Basidiomycota these were classes Agaricomycetes (families *Glomeraceae*, *Serendipitaceae*, *Russulaceae* in both, *Sebacinaceae* and *Inocybaceae* only in tank water), and Tremellomycetes (*Piskurozymaceae* in cloaca). In Glomeromycota and Mortierellomycota, classes Glomeromycetes (family *Glomeraceae*) and Mortierellomycetes (family *Mortierellaceae*) were present above 1% average RA in both cloaca and tank water, respectively (Table S4). Out of all fungal ASVs, 18% could not be identified below phylum level (Table S4).

Clinically relevant taxa such as *Fusarium* spp. (family *Nectriaceae*), *Cladosporium* spp. (family *Davidiellaceae*), and *Penicillium* spp. (family *Trichocomaceae*) were detected sporadically across samples at or around 1-2% RA, with *Fusarium* spp. reaching 8% RA in ITS0094C sample. Two ASVs assigned to *Nectriaceae* and *Solicoccozyma aeria* were shared across 90% of all samples (26/29), while 90% of cloacal samples (18/20) shared an ASV assigned to *Nectriaceae*, and 100% of tank water samples (9/9) shared eight ASVs assigned to *Nectriaceae*, *Bartaliniaceae*, *Serendipitaceae*, *Clonostachys* sp., *Tetracladium* sp., *Solicoccozyma aeria*, *Ilyonectria robusta*, and *Pseudaleuria* sp. (Table S4).

Negative control sample yielded 60,329 ITS2 reads (with 1,131 fungal ASVs) most of which belonged to Ascomycota (90%) (Table S4). Out of all negative control fungal ASVs 424 were detected in cloacal and tank water samples making up 71% of total reads in negative control, 36% in cloacal and 32% of total reads in tank water samples. Maximum RA of an individual ASV in negative control reached 2% (assigned to *Inocybe maculata*), with top 14 ASVs at 1-2% RA.

Epizoic and endozoic bacterial communities

The carapace samples mostly consisted of families *Pseudoalteromonadaceae* (*Pseudoalteromonas* spp.), *Rhodobacteraceae* (unclassified and *Paracoccus* spp.), *Flavobacteriaceae* (unclassified and *Tenacibaculum* spp.), *Vibrionaceae* (*Vibrio* spp.), *Alteromonadaceae* (*Alteromonas* spp.), *Moraxellaceae* (*Acinetobacter* spp., *Psychrobacter* spp.), *Saccharospirillaceae* (unclassified and *Oceaniserpentilla* spp.), *Arcobacteraceae*, *Sphingomonadaceae*, *Colwelliaceae* (*Colwellia* spp.),

Cyclobacteriaceae, *Shewanellaceae* (*Shewanella* spp.), *Spongiibacteraceae* (BD1-7 clade), *Cellvibrionaceae*, JGI 0000069-P22, and *Pseudomonadaceae* (*Pseudomonas* spp.) (Table S5).

Differential abundance analysis on taxa collapsed to species level (structural zeros excluded) detected 58 DA taxa (Fig. S2). Most taxa were found to be DA in carapace (*Erythrobacter* spp., *Thalassospira* spp., *Leptolynbya* sp., etc.) or tank water samples (e.g., *Cryomorphaceae* spp., NS3a marine group, *Marinomonas* spp.). Taxa collapsed to *Tenacibaculum*, *Moraxellaceae*, *Cardiobacteriaceae*, *Marinifilum*, *Campylobacter*, and Bacteroidales, were consistently DA in both cloaca and oral cavity in comparison to carapace and tank water. Bacteroides, WCHB1.41, *Rikenellaceae* RC9 gut group, *Cardiobacterium*, *Shewanella* were consistent in cloacal samples, while *Halioglobus* and *Truepera* were DA in oral samples (Fig. S2).

SUPPLEMENTARY REFERENCES

1. Filek K, Trotta A, Gračan R, Di Bello A, Corrente M, Bosak S (2021) Characterization of oral and cloacal microbial communities of wild and rehabilitated loggerhead sea turtles (Caretta caretta). Anim Microbiome 3:59. https://doi.org/10/gmptgh

2. Mariani G, Bellucci F, Cocumelli C, Raso C, Hochscheid S, Roncari C, Nerone E, Recchi S, Di Giacinto F, Olivieri V, Pulsoni S, Matiddi M, Silvestri C, Ferri N, Renzo LD (2023) Dietary Preferences of Loggerhead Sea Turtles (Caretta caretta) in Two Mediterranean Feeding Grounds: Does Prey Selection Change with Habitat Use throughout Their Life Cycle? Anim Open Access J MDPI 13:654. https://doi.org/10.3390/ani13040654

3. Bolyen E, Rideout JR, Dillon MR, Bokulich NA, Abnet CC, Al-Ghalith GA, Alexander H, Alm EJ, Arumugam M, Asnicar F, Bai Y, Bisanz JE, Bittinger K, Brejnrod A, Brislawn CJ, Brown CT, Callahan BJ, Caraballo-Rodríguez AM, Chase J, Cope EK, Da Silva R, Diener C, Dorrestein PC, Douglas GM, Durall DM, Duvallet C, Edwardson CF, Ernst M, Estaki M, Fouquier J, Gauglitz JM, Gibbons SM, Gibson DL, Gonzalez A, Gorlick K, Guo J, Hillmann B, Holmes S, Holste H, Huttenhower C, Huttley GA, Janssen S, Jarmusch AK, Jiang L, Kaehler BD, Kang KB, Keefe CR, Keim P, Kelley ST, Knights D, Koester I, Kosciolek T, Kreps J, Langille MGI, Lee J, Ley R, Liu YX, Loftfield E, Lozupone C, Maher M, Marotz C, Martin BD, McDonald D, McIver LJ, Melnik AV, Metcalf JL, Morgan SC, Morton JT, Naimey AT, Navas-Molina JA, Nothias LF, Orchanian SB, Pearson T, Peoples SL, Petras D, Preuss ML, Pruesse E, Rasmussen LB, Rivers A, Robeson MS, Rosenthal P, Segata N, Shaffer M, Shiffer A, Sinha R, Song SJ, Spear JR, Swafford AD, Thompson LR, Torres PJ, Trinh P, Tripathi A, Turnbaugh PJ, Ul-Hasan S, van der Hooft JJJ, Vargas F, Vázquez-Baeza Y, Vogtmann E, von Hippel M, Walters W, Wan Y, Wang M, Warren J, Weber KC, Williamson CHD, Willis AD, Xu ZZ, Zaneveld JR, Zhang Y, Zhu Q, Knight R, Caporaso JG (2019) Reproducible, interactive, scalable and extensible microbiome data science using QIIME 2. Nat Biotechnol 37:852–857. https://doi.org/10.1038/s41587-019-0209-9

4. Katoh K (2002) MAFFT: a novel method for rapid multiple sequence alignment based on fast Fourier transform. Nucleic Acids Res 30:3059–3066. https://doi.org/10.1093/nar/gkf436

5. Price MN, Dehal PS, Arkin AP (2010) FastTree 2 - Approximately maximum-likelihood trees for large alignments. PLoS ONE 5:. https://doi.org/10.1371/journal.pone.0009490

6. Bokulich NA, Kaehler BD, Rideout JR, Dillon M, Bolyen E, Knight R, Huttley GA, Gregory Caporaso J (2018) Optimizing taxonomic classification of marker-gene amplicon sequences with QIIME 2's q2-feature-classifier plugin. Microbiome 6:90–90. https://doi.org/10.1186/s40168-018-0470-z

7. Robeson MS, O'Rourke DR, Kaehler BD, Ziemski M, Dillon MR, Foster JT, Bokulich NA (2020) RESCRIPt: Reproducible sequence taxonomy reference database management for the masses. 2020.10.05.326504

8. Pruesse E, Quast C, Knittel K, Fuchs BM, Ludwig W, Peplies J, Glockner FO (2007) SILVA: a comprehensive online resource for quality checked and aligned ribosomal RNA sequence data compatible with ARB. Nucleic Acids Res 35:7188–7196. https://doi.org/10.1093/nar/gkm864

9. Nilsson RH, Larsson K-H, Taylor AFS, Bengtsson-Palme J, Jeppesen TS, Schigel D, Kennedy P, Picard K, Glöckner FO, Tedersoo L, Saar I, Kõljalg U, Abarenkov K (2019) The UNITE database for molecular identification of fungi: handling dark taxa and parallel taxonomic classifications. Nucleic Acids Res 47:D259–D264. https://doi.org/10.1093/nar/gky1022

10. Parada AE, Needham DM, Fuhrman JA (2016) Every base matters: assessing small subunit rRNA primers for marine microbiomes with mock communities, time series and global field samples. Environ Microbiol 18:1403–1414. https://doi.org/10.1111/1462-2920.13023

11. Apprill A, McNally S, Parsons R, Weber L (2015) Minor revision to V4 region SSU rRNA 806R gene primer greatly increases detection of SAR11 bacterioplankton. Aquat Microb Ecol 75:129–137. https://doi.org/10.3354/ame01753

12. Ebani VV (2023) Bacterial Infections in Sea Turtles. Vet Sci 10:333. https://doi.org/10.3390/vetsci10050333

SUPPLEMENTARY TABLES AND FIGURES

Fig. S1 Fungal communities' diversity. Unweighted UniFrac PCoA plot and rAitchison PCA biplot
Fig. S2 Relative abundance of differentially abundant taxa for epizoic, endozoic and tank water V4 reads
Table S1Extended metadata on all samples in this study
Table S2 Clinical considerations for turtles in this study
Table S3 Raw read counts for endozoic and tank water samples V34
Table S4 Raw read counts for cloacal and tank water samples ITS2
Table S5 Raw read counts for epizoic, endozoic, and tank water samples V4

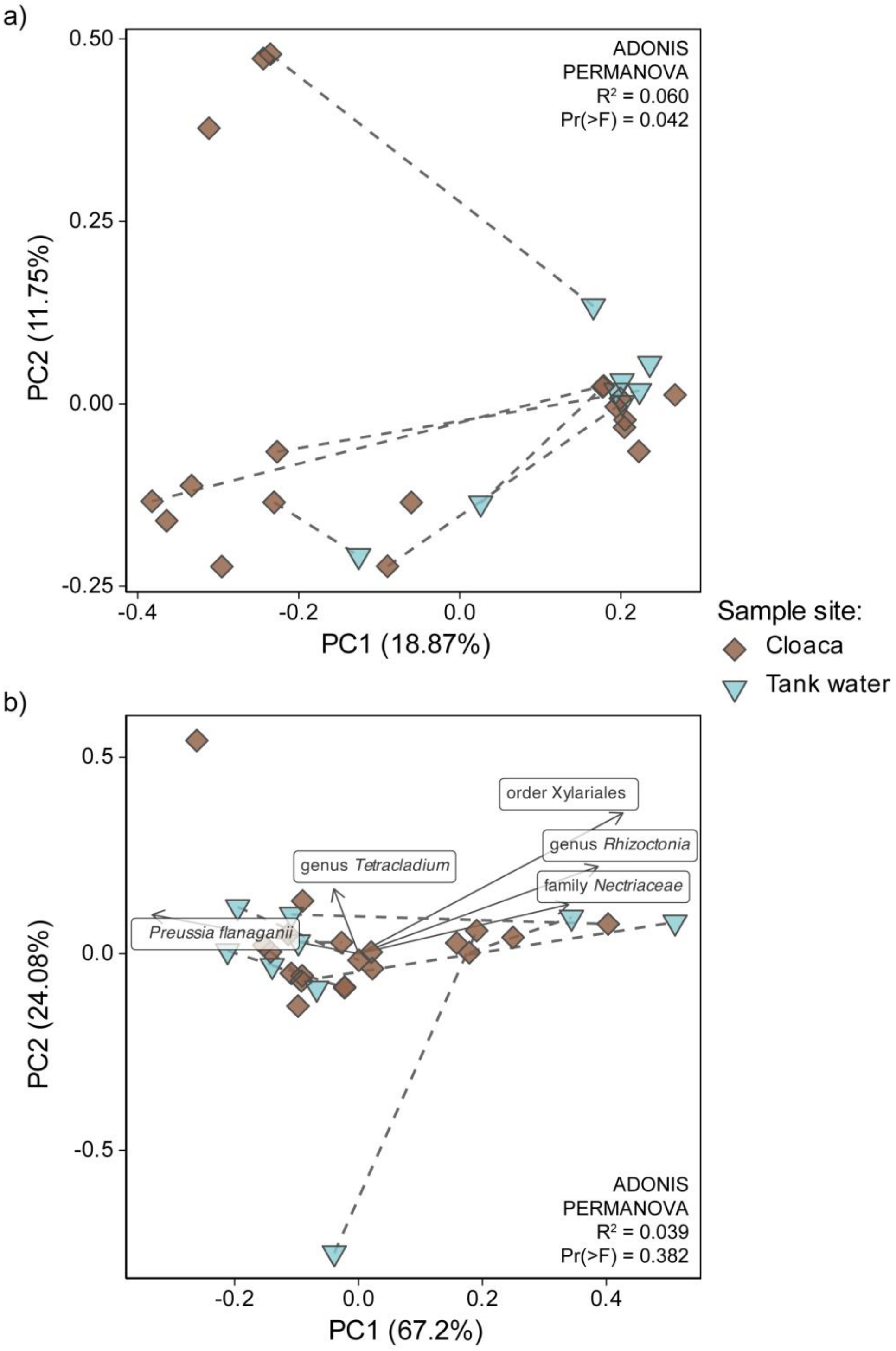

Fig. S1 Diversity of fungal communities in cloacal and tank water samples. (a) Unweighted UniFrac PCoA (a) and robust Aitchison PCA biplot (b) with loadings as highly ranked features. Corresponding cloacal and tank water samples are connected with dashed lines.

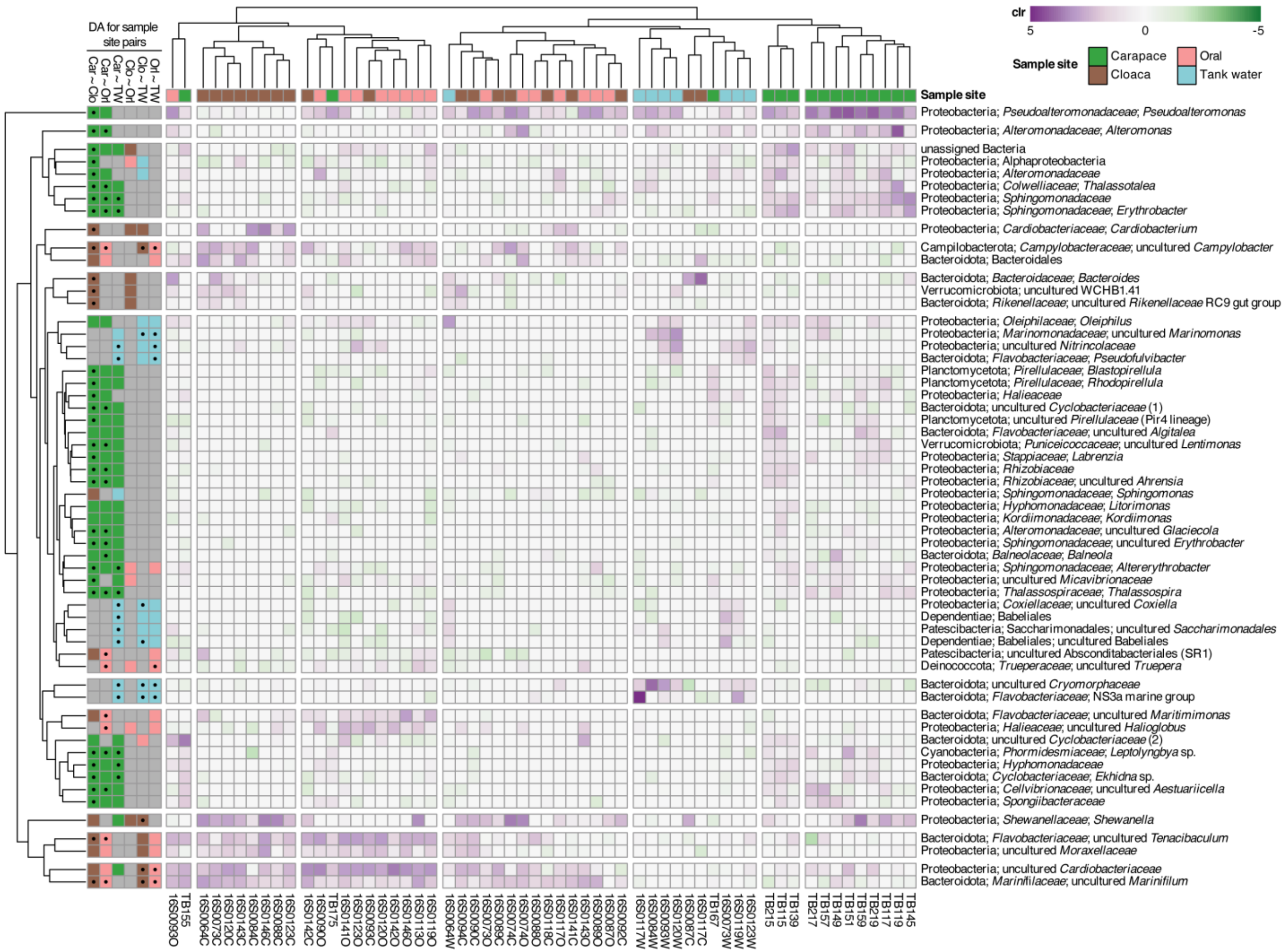

Fig. S2 Relative abundance [centered log ratio (clr)] of differentially abundant taxa (ANCOM-BC2, with structural zeros excluded) collapsed at species level in carapace, cloacal, oral and tank water samples. On the left side, the panel indicates in which sample site out of all sample site pairs the specific taxon is significantly differentially abundant (p-value < 0.05). Black dots indicate significance after correction for multiple testing (q-value < 0.05). The rows and columns were clustered using complete linkage method.
