# Supplementary material for: Loggerhead Sea Turtles as Hosts of Diverse Bacterial and Fungal Communities
Source: Microb Ecol. 2024 May 30;87:79. doi: 10.1007/s00248-024-02388-x (PMC11139726; doi:10.1007/s00248-024-02388-x)
Supplement: Supplementary file 2 [file 248_2024_2388_MOESM3_ESM.pdf]

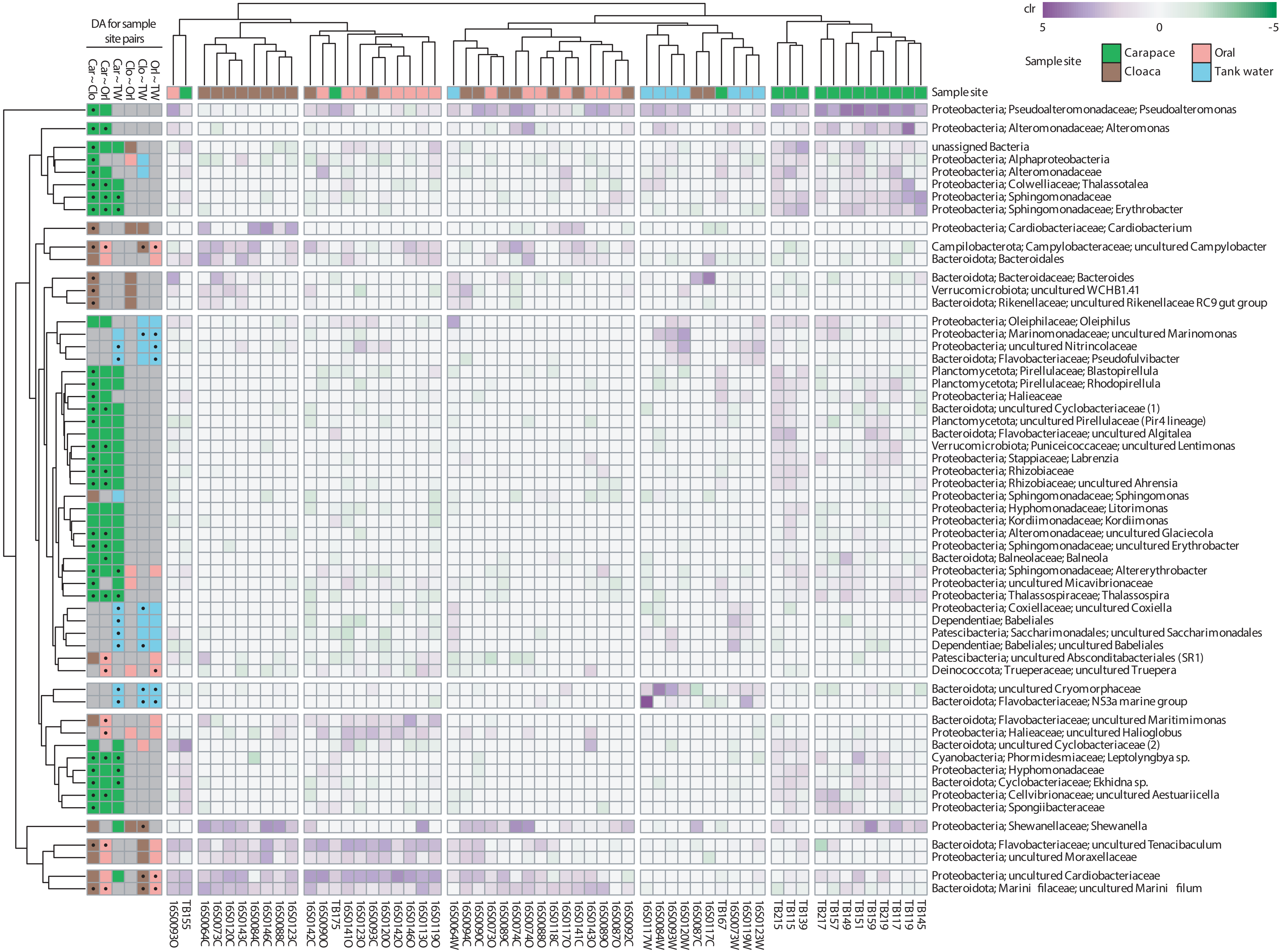

DA for sample site pairs
Car ~ Clo
Car ~ Orl
Car ~ TW
Clo ~ Orl
Clo ~ TW
Orl ~ TW
clr
5
0
-5
Sample site
Carapace
Cloaca
Oral
Tank water
Sample site
Proteobacteria; Pseudoalteromonadaceae; Pseudoalteromonas
Proteobacteria; Alteromonadaceae; Alteromonas
unassigned Bacteria
Proteobacteria; Alphaproteobacteria
Proteobacteria; Alteromonadaceae
Proteobacteria; Colwelliaceae; Thalassotalea
Proteobacteria; Sphingomonadaceae
Proteobacteria; Sphingomonadaceae; Erythrobacter
Proteobacteria; Cardiobacteriaceae; Cardiobacterium
Campilobacterota; Campylobacteraceae; uncultured Campylobacter
Bacteroidota; Bacteroidales
Bacteroidota; Bacteroidaceae; Bacteroides
Verrucomicrobiota; uncultured WCHB1.41
Bacteroidota; Rikenellaceae; uncultured Rikenellaceae RC9 gut group
Proteobacteria; Oleiphilaceae; Oleiphilus
Proteobacteria; Marinomonadaceae; uncultured Marinomonas
Proteobacteria; uncultured Nitrincolaceae
Bacteroidota; Flavobacteriaceae; Pseudofulvibacter
Planctomycetota; Pirellulaceae; Blastopirellula
Planctomycetota; Pirellulaceae; Rhodopirellula
Proteobacteria; Halieaceae
Bacteroidota; uncultured Cyclobacteriaceae (1)
Planctomycetota; uncultured Pirellulaceae (Pir4 lineage)
Bacteroidota; Flavobacteriaceae; uncultured Algitalea
Verrucomicrobiota; Puniceicoccaceae; uncultured Lentimonas
Proteobacteria; Stappiaceae; Labrenzia
Proteobacteria; Rhizobiaceae
Proteobacteria; Rhizobiaceae; uncultured Ahrensia
Proteobacteria; Sphingomonadaceae; Sphingomonas
Proteobacteria; Hyphomonadaceae; Litorimonas
Proteobacteria; Kordiimonadaceae; Kordiimonas
Proteobacteria; Alteromonadaceae; uncultured Glaciecola
Proteobacteria; Sphingomonadaceae; uncultured Erythrobacter
Bacteroidota; Balneolaceae; Balneola
Proteobacteria; Sphingomonadaceae; Altererythrobacter
Proteobacteria; uncultured Micavibrionaceae
Proteobacteria; Thalassospiraceae; Thalassospira
Proteobacteria; Coxiellaceae; uncultured Coxiella
Dependentiae; Babeliales
Patescibacteria; Saccharimonadales; uncultured Saccharimonadales
Dependentiae; Babeliales; uncultured Babeliales
Patescibacteria; uncultured Absconditabacteriales (SR1)
Deinococcota; Trueperaceae; uncultured Truepera
Bacteroidota; uncultured Cryomorphaceae
Bacteroidota; Flavobacteriaceae; NS3a marine group
Bacteroidota; Flavobacteriaceae; uncultured Maritimimonas
Proteobacteria; Halieaceae; uncultured Haliglobus
Bacteroidota; uncultured Cyclobacteriaceae (2)
Cyanobacteria; Phormidesmiaceae; Leptolyngbya sp.
Proteobacteria; Hyphomonadaceae
Bacteroidota; Cyclobacteriaceae; Ekhidna sp.
Proteobacteria; Cellvibrionaceae; uncultured Aestuariicella
Proteobacteria; Spongiibacteraceae
Proteobacteria; Shewanellaceae; Shewanella
Bacteroidota; Flavobacteriaceae; uncultured Tenacibaculum
Proteobacteria; uncultured Moraxellaceae
Proteobacteria; uncultured Cardiobacteriaceae
Bacteroidota; Marini filaceae; uncultured Marini filum
16S0093O
TB155
16S0064C
16S0073C
16S0120C
16S0143C
16S0084C
16S0146C
16S0088C
16S0123C
16S0142C
16S0090O
TB175
16S0141O
16S0123O
16S0093C
16S0120O
16S0142O
16S0146O
16S0113O
16S0119O
16S0064W
16S0094C
16S0090C
16S0073O
16S0089C
16S0074C
16S0074O
16S0088O
16S0118C
16S0117O
16S0141C
16S0143O
16S0089O
16S0087O
16S0092C
16S0117W
16S0084W
16S0093W
16S0120W
16S0087C
16S0117C
TB167
16S0073W
16S0119W
16S0123W
TB215
TB115
TB139
TB217
TB157
TB149
TB151
TB159
TB219
TB117
TB119
TB145
