# Supplementary figures and images for: Loggerhead Sea Turtles as Hosts of Diverse Bacterial and Fungal Communities
Source: Microb Ecol. 2024 May 30;87(1):79. doi: 10.1007/s00248-024-02388-x (PMC11139726; doi:10.1007/s00248-024-02388-x)

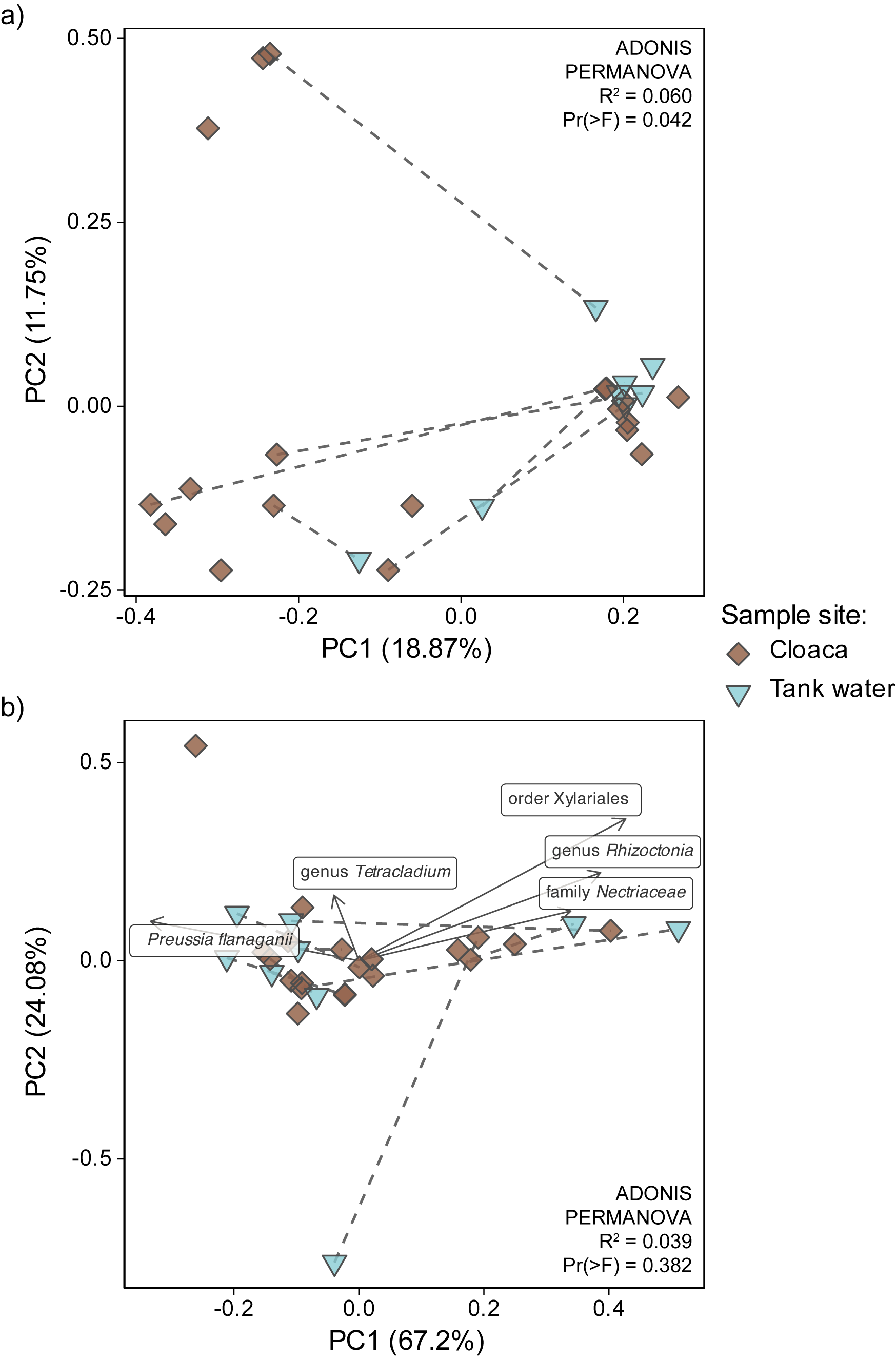

Supplement: Supplementary file 2 — Supplementary Material 2 [file 248_2024_2388_MOESM2_ESM.png]

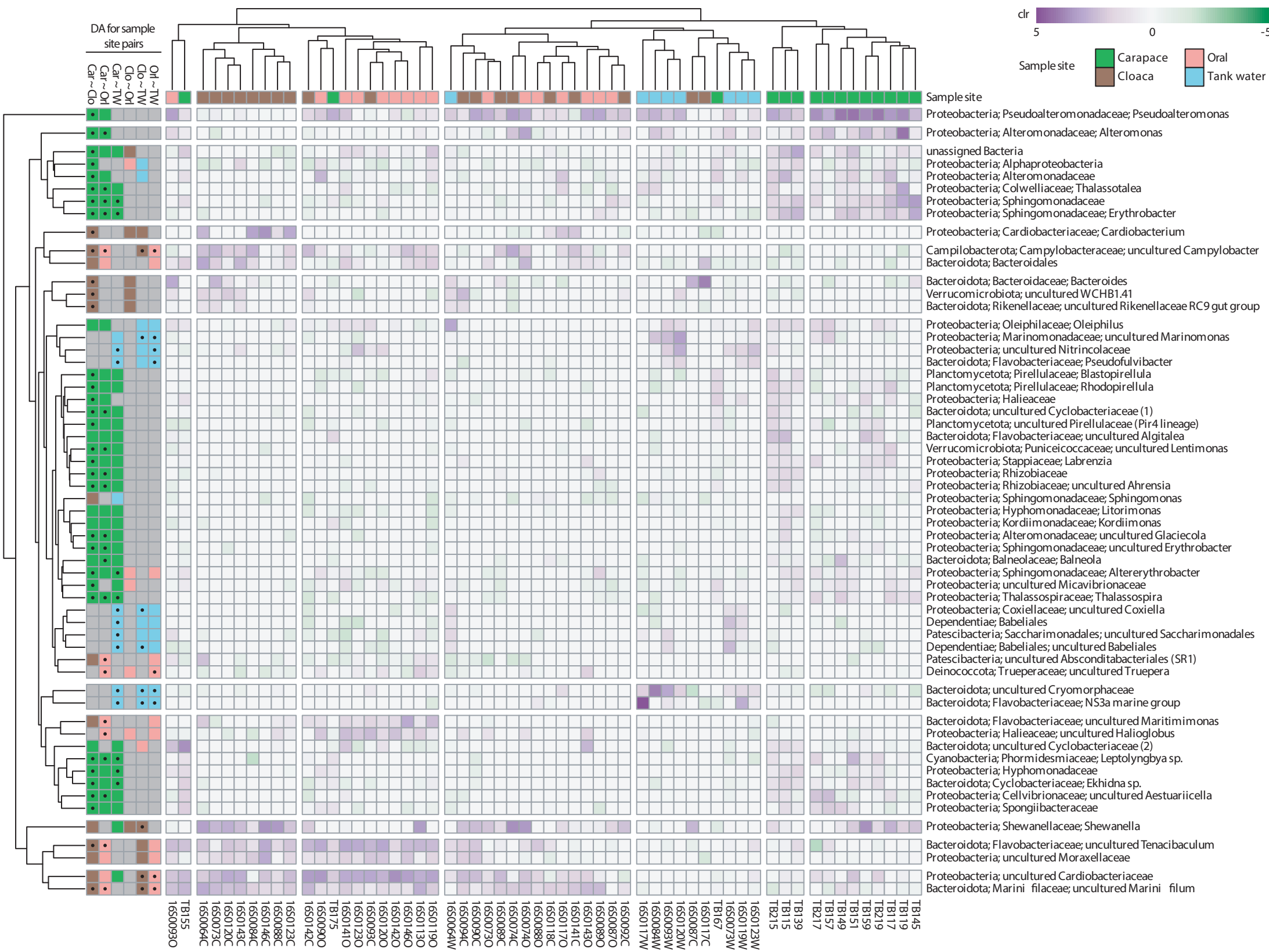

Supplement: Supplementary file 3 — Supplementary Material 3 [file 248_2024_2388_MOESM3_ESM.pdf]
